# When digital engagement becomes dysregulated: associations with aggression, achievement motivation, and academic performance in adolescents

**DOI:** 10.3389/fpubh.2026.1896602

**Published:** 2026-09-03

**Authors:** Minghua Dong, Huitao He, Bowen Wei

**Affiliations:** 1 Suzhou Institute of Trade & Commerce, Suzhou, China; 2 School of Business, Soochow University, Suzhou, China; 3 School of Labor and Human Resources, Renmin University of China, Beijing, China

**Keywords:** academic performance, achievement motivation, aggression, dysregulated digital engagement, self-regulation

## Abstract

**Introduction:**

Adolescents are embedded in digital environments that are increasingly central to social, academic, and emotional life. Yet the developmental implications of digital engagement remain contested, particularly when exposure metrics such as screen time are used as primary indicators. The present study examined digital engagement as a multidimensional construct while treating regulatory difficulty—the dimension most directly reflecting impaired control and difficulty disengaging—as the focal predictor of aggression, achievement motivation, and objectively measured academic performance.

**Methods:**

Participants were 309 secondary school students in eastern China (Mage = 14.52 years) who completed self-report measures of dysregulated digital engagement, multidimensional aggression, and achievement motivation; official semester GPA was obtained from school records. Confirmatory factor analyses were conducted to evaluate the measurement structure of the scales. Correlational analyses and classroom-cluster-adjusted hierarchical modeling were used to examine associations between regulatory difficulty and the study outcomes while accounting for demographic and design-related covariates, total aggression, and achievement motivation.

**Results:**

Confirmatory factor analyses supported the measurement structure of all scales. Correlational analyses indicated that dysregulated engagement was positively associated with aggression and negatively associated with achievement motivation and GPA. In the classroom-cluster-adjusted hierarchical model, regulatory difficulty showed a modest incremental negative association with GPA after demographic and design-related covariates, total aggression, and achievement motivation were statistically accounted for.

**Discussion:**

Findings are consistent with a self-regulation perspective and indicate that dysregulated characteristics of digital engagement are meaningfully associated with adolescent academic functioning. Because digital exposure duration was not independently assessed, the present findings should be interpreted as complementing, rather than directly contrasting with, screen-time research. These findings highlight the potential value of focusing on digital self-regulation, rather than exposure duration alone, in future research and educational practice.

## 1 Introduction

Adolescence unfolds within an unprecedentedly digitized sociocognitive ecology. Over the past two decades, digital environments have moved from peripheral tools of communication to structurally embedded components of adolescents’ everyday lives. For contemporary youth, online platforms are not merely channels for information exchange; they function as social arenas, identity laboratories, entertainment ecosystems, and increasingly educational infrastructures. This profound integration of digital technologies into daily routines has generated an urgent scientific question: under what conditions does digital engagement support adolescent development, and when does it begin to undermine behavioral regulation, motivational processes, and academic functioning? Early research on youth and media relied heavily on exposure metrics, particularly time spent online. While screen time remains an intuitively appealing index, accumulating evidence suggests that duration alone provides an incomplete account of digital influence (1, 2). Large-scale investigations indicate that simple time-based measures often show small effect sizes and may obscure substantial heterogeneity in usage patterns (3). Umbrella reviews and integrative analyses similarly emphasize that digital engagement effects are context-dependent and vary across adolescents (4, 5). These findings have encouraged increasing attention to characteristics of digital engagement that are not captured by duration alone, including the context, purpose, intensity, and degree of perceived control over digital behavior. Evidence from Chinese youth further supports this differentiated perspective: Jiang et al. (6) found that Internet-use duration, purpose of use, and dimensions of Internet literacy were differentially associated with Internet addiction, with Internet self-management showing an inverse association with addiction risk. In parallel with these debates, a differentiated conceptualization of digital engagement has emerged, emphasizing qualitative dimensions such as emotional investment, compulsive tendencies, and regulatory difficulty (7–9). Within this framework, digital behavior becomes consequential not merely when frequent, but when dysregulated. Related evidence from gaming contexts suggests that continued digital engagement can persist despite adverse subjective experiences: Zhang et al. (10) found that obsessive passion was associated with greater gaming burnout and lower quitting intentions, with highly obsessive players remaining less likely to disengage even when experiencing burnout. Dysregulated digital engagement refers to patterns characterized by diminished self-control, difficulty disengaging, and interference with daily responsibilities (11, 12). Studies linking problematic smartphone use to academic and psychological outcomes suggest that regulatory difficulty represents an additional dimension of digital behavior that warrants examination alongside conventional exposure indicators.

### 1.1 Digital engagement and self-regulation

Adolescence represents a particularly sensitive developmental window for examining these processes. Heightened sensitivity to reward and social evaluation may amplify responsiveness to digitally mediated reinforcement systems. Social media platforms are intentionally designed around intermittent reinforcement and social validation mechanisms, features known to strengthen habitual responding (13). Emerging neuroscientific and behavioral research indicates that adolescents may be especially responsive to such reward structures, increasing the potential for dysregulated engagement (14, 15). Recent evidence further indicates that digital-use patterns are embedded in affective and control-related processes: Chu et al. (16) found that short- and long-term negative affect showed distinct associations with smartphone use and that locus of control moderated these relationships.

In the present study, self-regulation is used as a theoretical perspective rather than as a directly measured construct. Broadly, self-regulation refers to the capacity to modulate attention, behavior, motivation, and goal-directed action in accordance with longer-term demands and goals. This perspective is relevant to digital engagement because difficulty disengaging from highly salient activities may coexist with difficulties sustaining goal-directed behavior in other domains. Consistent with the broader relevance of self-regulatory capacities in digital educational environments, Huang et al. (17) found that students’ self-regulated learning moderated the associations between social support received in online discussion forums and dimensions of online learning burnout. Previous research has associated problematic or poorly regulated digital use with attentional disruption, procrastination, and lower academic productivity (18, 19), while meta-analytic and review evidence has linked excessive or problematic digital engagement with modest differences in academic functioning (20, 21). However, the present study does not directly assess general self-regulatory capacity, executive control, or attentional regulation. Accordingly, self-regulation serves only as an interpretive framework for organizing the examined constructs and should not be understood as a mechanism empirically tested by the present data. Aggression and achievement motivation were selected as theoretically informative correlates because they represent two distinct domains of adolescent functioning with direct relevance to school adjustment.

### 1.2 Aggression, achievement motivation, and academic functioning

Aggression was conceptualized according to the multidimensional framework of Buss and Perry (22), which distinguishes behavioral, affective, and cognitive components. Physical Aggression and Verbal Aggression represent behavioral expressions of aggression, Anger reflects its affective component, and Hostility captures the cognitive component involving negative or antagonistic appraisals. In the present study, total aggression was treated as the primary broad aggression construct, whereas the four dimensions were examined separately only in exploratory analyses. This distinction is important because the study assesses general aggressive tendencies rather than specifically online or digitally expressed aggression. Prior research has reported associations between problematic forms of digital engagement and greater aggressive tendencies, although their direction and underlying mechanisms remain uncertain (23, 24). Aggression is also relevant to academic functioning because disruptive behavior, interpersonal conflict, and emotional reactivity may be associated with poorer classroom adjustment and learning.

Achievement motivation represents a complementary motivational domain. Academic success requires persistence, effort investment, and sustained commitment to goals whose rewards are often delayed (25, 26). Recent evidence from Chinese higher-education settings further illustrates the broader relevance of motivational beliefs to academic functioning: Tang et al. (27) identified multiple configurations of teacher caring and academic self-efficacy associated with high academic achievement, underscoring the multifactorial nature of academic performance. Dysregulated digital engagement may coexist with lower levels of these motivational characteristics, particularly when digital activities interfere with goal-directed behavior or compete with sustained academic engagement. Existing research has reported associations between problematic digital use and reduced academic productivity and performance (20, 28), although such evidence does not establish causal direction.

In the present study, aggression and achievement motivation are therefore not conceptualized as mediators through which digital engagement necessarily affects academic performance, nor are they treated as direct measures of self-regulatory capacity. Rather, they are examined as theoretically relevant concurrent correlates and as established behavioral and motivational predictors of academic performance. Including them in the same analysis allows a more focused question to be addressed: whether dysregulated digital engagement retains an association with objectively recorded academic performance after accounting for variance associated with these two domains. Self-regulation provides a theoretical lens for interpreting potential commonalities among these constructs, but self-regulation itself was not directly measured and its underlying mechanisms are not tested in the present study.

### 1.3 Study rationale and contribution

The distinction between exposure to digital media and difficulty regulating digital engagement is not itself novel; related distinctions are well established in research on problematic smartphone use, compulsive internet use, and other forms of dysregulated digital behavior. The contribution of the present study is therefore more specific. First, regulatory difficulty is examined separately from the frequency and intensity dimensions of digital engagement rather than being treated as interchangeable with general exposure. Second, regulatory difficulty is examined in relation to both multidimensional aggression and achievement motivation within the same adolescent sample. Third, academic performance is indexed using school-recorded GPA, allowing the incremental association of regulatory difficulty with an objectively recorded educational outcome to be examined after demographic and design-related covariates, total aggression, and achievement motivation are taken into account. The study also evaluates whether this GPA association persists when regulatory-difficulty items containing explicit academic-interference content are removed. Relevant research has previously been conducted in China, including studies of mobile-phone addiction and school performance (29) and social-media use and academic performance (30). Accordingly, the Chinese setting is treated as the context of the present study rather than as the source of its theoretical novelty. The study does not claim a uniquely Chinese mechanism or a cross-cultural effect. The study also does not directly compare dysregulated engagement with screen time and does not treat self-regulation as a measured mechanism. Instead, self-regulation serves as a theoretical perspective for considering why difficulty controlling digital engagement may coexist with behavioral and motivational characteristics relevant to academic functioning. The principal empirical objective is to determine whether dysregulated digital engagement shows an incremental association with school-recorded GPA after aggression and achievement motivation are statistically accounted for. This approach is intended to clarify concurrent and incremental relationships among the measured constructs without implying causal, cultural, or mechanistic effects that cannot be established from the present cross-sectional design.

## 2 The present study

The present study examined the concurrent associations among dysregulated digital engagement, aggression, achievement motivation, and objectively recorded academic performance in adolescents. Regulatory difficulty within digital engagement was treated as the focal predictor because it most directly captures perceived difficulty disengaging, impaired control, and interference with responsibilities. Frequency and intensity were retained as related but conceptually distinct dimensions and were examined in secondary analyses rather than being automatically combined with regulatory difficulty into the principal predictor. Aggression and achievement motivation were examined as theoretically relevant behavioral and motivational correlates that are also associated with school functioning. Self-regulation provided a theoretical interpretive perspective, but it was not directly measured and therefore was not modeled as a mediator or mechanism.

The study had two primary objectives. The first was to characterize the associations of dysregulated digital engagement with aggression and achievement motivation. The second, and principal analytic objective, was to determine whether regulatory difficulty in digital engagement accounted for additional variation in school-recorded GPA after total aggression and achievement motivation were included in the model. Frequency and intensity were examined separately in secondary analyses to determine whether the observed pattern was specific to regulatory difficulty or shared across the broader dimensions of digital engagement. Analyses involving specific aggression dimensions were treated as secondary and exploratory rather than as separate primary hypotheses.

Based on the literature reviewed above, the following hypotheses were specified:

*H1*: Higher regulatory difficulty in digital engagement would be positively associated with total aggression.

*H2*: Higher regulatory difficulty in digital engagement would be negatively associated with achievement motivation.

*H3*: Achievement motivation would be positively associated with GPA, whereas total aggression would be negatively associated with GPA.

*H4*: Regulatory difficulty in digital engagement would show a significant incremental negative association with GPA after total aggression, achievement motivation, and the demographic and design-related covariates were statistically accounted for.

Associations involving the individual aggression dimensions—Physical Aggression, Verbal Aggression, Anger, and Hostility—were treated as exploratory analyses. These analyses were intended to characterize the distribution of the overall engagement–aggression association across aggression domains rather than to test separate primary hypotheses. No directional hypothesis concerning the relative strength of these subdimension associations was specified.

Because the study used a cross-sectional design, all hypotheses concern statistical associations rather than temporal or causal effects. Likewise, the study does not test whether dysregulated engagement is more predictive than screen-time exposure, because duration of digital use was not independently assessed. The primary analyses therefore focus specifically on the regulatory-difficulty dimension, which most directly corresponds to the theoretical construct of dysregulated engagement. Frequency and intensity are examined separately as secondary dimensions, allowing their associations to be described without assuming that all three dimensions form a single interchangeable total construct.

## 3 Method

### 3.1 Participants

Participants were 316 adolescents (158 girls, 158 boys) recruited from three public secondary schools located in an urban district of eastern China. Schools were selected to reflect typical mid-sized public institutions serving mixed socioeconomic populations. Students ranged in age from 13 to 16 years (*M* = 14.52, SD = 0.94), corresponding to Grades 7 through 9 within the Chinese compulsory education system. The recruited sample was approximately balanced by gender, although gender was not a primary focus of the study and was included as a demographic covariate in the adjusted GPA analysis. Cluster sampling was employed at the classroom level. Within each participating school, intact classes were randomly selected from grade rosters, and all students in selected classrooms were invited to participate. The analytic sample was distributed across 12 intact classrooms from the three participating schools, with four classrooms contributed by each school. Classroom analytic sample sizes ranged from 24 to 27 students (*M* = 25.75, SD = 1.14). Because students were nested within classrooms and classrooms were nested within schools, this sampling structure was explicitly considered in the revised analyses. Classroom-level intraclass correlation coefficients (ICCs) were estimated for GPA and the principal study variables before regression modelling. School membership and grade level were retained as design-related covariates for the adjusted GPA models because both could capture systematic differences in instructional context and grading practices. Written informed consent was obtained from parents or legal guardians, and assent was obtained from all participating students prior to data collection. Participation was voluntary, and students were informed that they could discontinue at any time without academic consequence. Exclusion criteria included self-reported history of neurological disorders, severe psychiatric diagnoses, or learning disabilities that could substantially impair comprehension of questionnaire items. These criteria were assessed through a brief confidential screening form administered at the beginning of the survey session. Four students met exclusion criteria and were removed prior to statistical analysis. Subsequently, data screening procedures were conducted to identify influential or extreme cases. Multivariate outliers were examined using Mahalanobis distance with a conservative threshold of *p* < 0.001. Three additional cases exceeded this criterion and were removed prior to hypothesis testing. All subsequent analyses were conducted using the final analytic sample of 309 adolescents. An independence-based power calculation aligned with the revised final model was conducted in G*Power using the test “Linear multiple regression: Fixed model, *R*^2^ increase.” The focal test concerned the incremental contribution of regulatory difficulty after the demographic/design-related covariates and comparison predictors had already been entered. Regulatory difficulty was therefore specified as one tested predictor, with eight predictors in the full model: age, gender, grade level, two school-indicator variables, total aggression, achievement motivation, and regulatory difficulty. Using *α* = 0.05, statistical power of 0.80, and an incremental effect size of Cohen’s *f*^2^ = 0.15, defined as *f*^2^ = ΔR^2^/(1 − R^2^full), the independence-based calculation indicated a minimum required sample of approximately 55 participants. However, this calculation assumes independent observations and therefore does not incorporate the loss of statistical efficiency associated with classroom clustering. Accordingly, the nominal sample-size calculation was not used to claim adequate power for small incremental effects; instead, the precision of the observed estimates was evaluated using the small-sample-corrected classroom-clustered inferential framework described below.

### 3.2 Procedure

Data collection was conducted during regular school hours in coordinated sessions scheduled with school administrators. Trained graduate research assistants administered the survey instruments in classroom settings under standardized instructions. Students were seated apart to ensure privacy and were instructed not to discuss responses with peers. The full survey required approximately 35–40 min to complete. To reduce common method bias, several procedural safeguards were implemented. First, scale sections were presented in counterbalanced order across classrooms to minimize systematic order effects. Second, response anchors were not identical across all instruments, which reduced the uniformity of response framing across scale sections. Third, students were assured that responses were confidential and would not be accessible to teachers or parents, thereby reducing social desirability pressures. Academic performance data were obtained from official school records following completion of the survey phase. Each student was assigned an anonymized identification code that allowed questionnaire responses to be matched with semester grade point average (GPA) without revealing personal identifiers. GPA values reflected standardized semester averages across core academic subjects (Chinese, mathematics, English, science, and social studies), calculated on a 0–20 scale consistent with regional grading practices.

Ethical approval for the study was granted by the affiliated university’s Institutional Review Board. All procedures adhered to the ethical principles outlined in the Declaration of Helsinki.

### 3.3 Measures

The three self-report constructs were assessed using different measurement approaches. Aggression was assessed using a previously established multidimensional aggression instrument, whereas the digital-engagement and achievement-motivation measures were adapted for the present study from established conceptual and measurement traditions. Accordingly, the latter two instruments are described below as adapted measures rather than as fully standardized instruments. For transparency and reproducibility, the revised Supplementary material provides the item wording, item-to-dimension assignment, response format, scoring procedure, and identification of any reverse-scored items for the adapted measures. Additional measurement details, including item allocation and scoring procedures for the adapted instruments, are provided in Supplementary Table S1 to facilitate reproducibility.

### 3.4 Digital engagement

Dysregulated digital engagement was assessed using a 20-item adapted measure developed for the present study to capture three related dimensions: frequency of digital engagement, intensity of involvement, and regulatory difficulty. The digital-engagement instrument was a study-adapted measure rather than an unmodified administration of a single previously standardized scale. Its item pool was informed by established conceptual and measurement traditions in problematic internet and smartphone use, particularly constructs involving salience, impaired control, difficulty disengaging, repetitive engagement, and interference with everyday functioning (7, 8, 11, 12). Items were organized to represent three conceptually distinguishable dimensions: frequency of engagement, intensity of involvement, and regulatory difficulty. Supplementary Table S1 reports the complete 20-item set together with each item’s dimensional assignment, scoring direction, and conceptual adaptation basis.

The preliminary item pool was reviewed for relevance to adolescent digital behavior and comprehensibility within the school context. The items were translated into Chinese and independently back-translated into English, and discrepancies between the source and back-translated versions were resolved through comparison of the two versions before administration. Participants responded using a five-point Likert scale ranging from 1 (strongly disagree) to 5 (strongly agree). Because the instrument was study adapted, its psychometric properties were evaluated in the present sample rather than assumed from an existing standardized scale. Internal consistency was examined for the relevant scores, and the theoretically specified three-factor structure was evaluated using confirmatory factor analysis. The present manuscript does not treat these procedures as equivalent to a separate formal content-validity study or independent external validation of the measure; accordingly, the findings are interpreted as supporting the measurement structure within the present sample rather than establishing definitive validation across populations.

Because the three dimensions are conceptually distinguishable, dimension-level results and the justification for any composite scoring are reported explicitly in the revised analyses rather than assuming that a three-factor solution alone establishes the validity of a single total score. For confirmatory factor analysis, the 20 items were specified *a priori* as indicators of three correlated first-order factors: frequency (6 items), intensity (6 items), and regulatory difficulty (8 items), following the item allocation reported in Supplementary Table S1. Each item was permitted to load only on its designated factor. No items were removed, no cross-loadings were estimated, and no correlated residuals were introduced. Factor covariances were freely estimated.

For the primary hypothesis tests, the regulatory-difficulty subscale (DE13–DE20) was used as the focal digital-engagement predictor because it most directly operationalizes the construct emphasized in the theoretical framework. Frequency and intensity scores were analyzed separately in secondary analyses. A 20-item overall score was not used as the principal predictor because evidence for a three-factor structure does not, by itself, establish the interpretability of a single total score. Because DE17–DE19 explicitly refer to homework, studying, or school responsibilities, a content-overlap sensitivity score was also calculated after excluding these three items. This reduced regulatory-difficulty score comprised DE13–DE16 and DE20, with DE20 reverse scored.

### 3.5 Aggressive behavior

Aggression was assessed using the 29-item Buss–Perry Aggression Questionnaire [BPAQ; (22)], which assesses four dimensions of aggression: Physical Aggression, Verbal Aggression, Anger, and Hostility. Participants rated each item using a five-point response format ranging from 1 (extremely uncharacteristic of me) to 5 (extremely characteristic of me). Items designated for reverse scoring were recoded before score calculation. Subscale scores were calculated by directly summing the items assigned to each BPAQ dimension: Physical Aggression (9 items), Verbal Aggression (5 items), Anger (7 items), and Hostility (8 items). Total aggression was calculated as the direct sum of all 29 BPAQ items after reverse scoring. No prorating or scale transformation was applied. Accordingly, the theoretical score ranges were 9–45 for Physical Aggression, 5–25 for Verbal Aggression, 7–35 for Anger, 8–40 for Hostility, and 29–145 for Total Aggression. Higher scores indicated greater aggression.

The four BPAQ dimensions were retained separately for exploratory dimension-level analyses, whereas the total aggression score was used in the primary GPA regression model. The confirmatory factor model followed the established four-factor structure of the BPAQ, with the 29 items assigned to Physical Aggression (9 items), Verbal Aggression (5 items), Anger (7 items), and Hostility (8 items) as specified in Supplementary Table S1. The four first-order factors were permitted to correlate. Each item loaded only on its designated factor; no item deletions, cross-loadings, or correlated residuals were specified. Internal consistency coefficients for the total scale and individual dimensions are reported in the Results section. The item composition, scoring direction, and subscale allocation are summarized in Supplementary Table S1.

### 3.6 Achievement motivation

Achievement motivation was assessed using a 28-item adapted measure designed to capture three closely related aspects of academic goal pursuit: persistence, effort investment, and task orientation. The instrument was informed by established work on academic motivation and self-determination (25, 26), but it was not treated as an unmodified administration of the Academic Motivation Scale. Accordingly, we refer to it as an adapted achievement-motivation measure rather than as a standardized instrument.

Participants responded on a five-point Likert scale ranging from 1 (strongly disagree) to 5 (strongly agree). Higher scores reflected greater achievement motivation after any negatively keyed items were reverse scored. The complete item wording, item-to-dimension allocation, and scoring procedure are provided in Supplementary Table S1. The confirmatory factor model specified three correlated first-order factors corresponding to persistence (10 items), effort investment (9 items), and task orientation (9 items), using the item assignments reported in Supplementary Table S1. All items loaded only on their designated factor. No items were removed, no cross-loadings were estimated, and no correlated residuals were included. Internal consistency and factorial structure were evaluated in the present sample and are reported in the Results section.

### 3.7 Academic performance

Academic performance was operationalized as official semester GPA obtained from school records. GPA reflected cumulative performance across core subjects and was calculated by school administrators according to standardized regional guidelines. In the final analytic sample (N = 309), mean GPA was 15.89 (SD = 2.09) on the 0–20 scale. The use of objective academic records strengthens validity and reduces reliance on self-report.

### 3.8 Data analytic strategy

All statistical analyses were conducted using SPSS 27 and AMOS 26. Prior to hypothesis testing, data were screened for missing values, distributional assumptions, multicollinearity, and influential cases. Missing data were minimal (<2% per variable) and handled using expectation–maximization procedures after Little’s MCAR test was nonsignificant, providing no evidence against the assumption that the data were missing completely at random. Normality was assessed through skewness and kurtosis indices, histograms, and Q–Q plots. The analyzed scale scores showed no substantial departures from normality, with skewness ranging from −0.41 to 0.88 and kurtosis ranging from −0.12 to 0.91, consistent with the descriptive statistics reported in Table 1. Confirmatory factor analyses were conducted separately for the three multidimensional self-report measures using correlated-factor simple-structure models specified according to the measurement structures described above. In each CFA, individual items were specified to load only on their designated first-order factor, factor covariances were freely estimated, and item residuals were specified as uncorrelated. No *post hoc* item deletions, cross-loadings, or residual covariances were introduced to improve model fit. Model fit was evaluated using the chi-square statistic, Comparative Fit Index (CFI), Tucker–Lewis Index (TLI), Root Mean Square Error of Approximation (RMSEA) with 90% confidence intervals, and Standardized Root Mean Square Residual (SRMR). Given that the analyzed variables were multi-item scale scores with distributions that showed no substantial departures from normality, Pearson correlations were used for the descriptive bivariate analyses. To evaluate the incremental association between dysregulated digital engagement and academic performance while accounting for the clustered sampling design, GPA was modelled using linear regression with classroom-clustered robust standard errors and a small-sample correction for inference. School and grade level were included as design-related covariates. Age and gender were also included in adjusted models because both were available for the full analytic sample and could plausibly covary with academic performance or the focal predictors.

**Table 1:** Descriptive statistics, reliability coefficients, and distributional indicators (*N* = 309).

Variable	*M*	SD	Min	Max	α	Skew	Kurtosis
Frequency	19.21	4.67	7	30	0.82	0.41	0.18
Intensity	19.29	4.85	7	30	0.84	0.48	0.26
Regulatory difficulty	24.62	6.20	10	39	0.86	0.57	0.44
Total aggression	72.44	14.11	41	108	0.91	0.71	0.49
Physical aggression	16.68	4.82	10	33	0.81	0.88	0.91
Verbal aggression	19.76	5.05	8	25	0.84	0.52	0.32
Anger	18.59	4.97	8	32	0.83	0.67	0.54
Hostility	17.41	4.73	9	34	0.79	0.73	0.61
Achievement motivation	94.07	12.98	60	125	0.88	−0.41	0.27
GPA	15.89	2.09	10.2	19.6	—	−0.36	−0.12

M = mean; SD = standard deviation; α = Cronbach’s alpha. BPAQ subscale and total-aggression scores were calculated by direct summation after reverse scoring of the designated items; no prorating or transformation was applied. The theoretical score ranges are 9–45 for Physical Aggression, 5–25 for Verbal Aggression, 7–35 for Anger, 8–40 for Hostility, and 29–145 for Total Aggression. Frequency, Intensity, and Regulatory Difficulty represent the three dimensions of the adapted digital-engagement measure. Regulatory Difficulty (DE13–DE20) was used as the focal digital-engagement predictor in the primary analyses, whereas Frequency and Intensity were examined separately in secondary analyses.

Predictors were entered hierarchically. The first block contained age, gender, grade, and school indicators. The second block added total aggression and achievement motivation. The third block added the regulatory-difficulty dimension of digital engagement. This ordering allowed the incremental contribution of digital engagement to be evaluated after both design/demographic covariates and the focal behavioral and motivational correlates had been taken into account. The analysis was not specified as a mediation or structural path model. Aggression and achievement motivation were treated as concurrent behavioral and motivational correlates rather than as intervening variables through which regulatory difficulty was assumed to influence academic performance. Given the cross-sectional design and the absence of a direct measure of the proposed self-regulatory mechanisms, indirect-effect estimates would not establish temporal or causal pathways. The hierarchical regression was therefore retained because it directly addressed the study’s narrower empirical question of whether regulatory difficulty showed an incremental association with GPA after the measured covariates, aggression, and achievement motivation were statistically accounted for. Unstandardized coefficients, standardized coefficients, cluster-robust standard errors, 95% confidence intervals, and adjusted significance tests were reported. Conventional R^2^ and ΔR^2^ values were retained as descriptive measures of explained variance, whereas statistical inference for individual regression coefficients was based on classroom-clustered robust standard errors with a small-sample correction. Variance Inflation Factors were inspected for multicollinearity.

The power calculation was formulated as an R^2^-increase problem because the principal question concerned the additional contribution of regulatory difficulty after the preceding predictors had been entered. Because the G*Power calculation assumed independent observations, it was treated only as an approximate reference calculation and not as evidence that the clustered sample provided adequate power for very small incremental effects. Inferential conclusions were therefore based on the small-sample-corrected classroom-clustered coefficient estimates, with R^2^ and ΔR^2^ reported descriptively.

Analyses were organized according to a revised hierarchy of evidential importance. Primary analyses examined the associations of regulatory difficulty with total aggression and achievement motivation and its incremental association with GPA. Secondary analyses examined the frequency and intensity dimensions of digital engagement separately. Exploratory analyses examined associations involving the four aggression dimensions—Physical Aggression, Verbal Aggression, Anger, and Hostility. The content-overlap analysis excluding DE17–DE19 from the regulatory-difficulty score was treated as a sensitivity analysis. This classification was used consistently when interpreting the results.

For the outcome-specific regression models, all variables were analyzed on their original scored metric rather than being rank transformed. Regulatory difficulty was entered as the single predictor in models for total aggression, achievement motivation, and the four aggression dimensions. Unstandardized and standardized coefficients were obtained from the same fitted models. Pearson coefficients were used for the descriptive bivariate analyses, whereas the regression coefficients were obtained directly from the corresponding raw-score linear models.

Statistical significance for the primary analyses was evaluated at *α* = 0.05. Because the four aggression-dimension analyses constituted a related family of exploratory tests, their *p* values were additionally evaluated using the Benjamini–Hochberg false-discovery-rate procedure. Unadjusted model estimates and confidence intervals were retained, and false-discovery-rate-adjusted significance decisions were reported for the exploratory aggression-dimension family. Secondary analyses involving frequency and intensity and the content-overlap sensitivity analysis were interpreted as supportive analyses and were not used to redefine the primary hypotheses.

Frequency and intensity were examined in separate secondary models rather than being combined with regulatory difficulty in the primary analysis. These analyses were intended to characterize whether associations with the study outcomes were specific to regulatory difficulty or were also present for more exposure-related dimensions of engagement. In addition, a content-overlap sensitivity analysis repeated the final GPA model after excluding DE17–DE19 from the regulatory-difficulty score because these items explicitly referred to homework, studying, or school responsibilities. Internal consistency of the resulting five-item reduced regulatory-difficulty score was estimated separately.

As a descriptive diagnostic of potential common method variance, Harman’s single-factor test was conducted across the self-report items. The first unrotated factor accounted for 27.4% of the total variance. Because this procedure cannot rule out shared method variance, the result was interpreted only as indicating that no single factor dominated the covariance among the self-report measures. Collectively, the analytic strategy was designed to quantify concurrent and incremental associations among the measured constructs while distinguishing primary, secondary, exploratory, and sensitivity analyses. The analyses were not intended to establish construct independence or to identify specific psychological mechanisms.

## 4 Results

### 4.1 Preliminary analyses

Prior to hypothesis testing, data were screened for accuracy, missing values, distributional properties, and multicollinearity. As described in the Participants section, data screening procedures resulted in a final analytic sample of 309 students. All subsequent analyses were conducted using this sample. Because participants were recruited in intact classrooms, classroom-level intraclass correlation coefficients (ICCs) were examined before hypothesis testing. The ICC for GPA was 0.047, indicating that approximately 4.7% of the variance in academic performance was attributable to between-classroom differences. Corresponding ICCs were 0.039 for regulatory difficulty, 0.032 for total aggression, and 0.044 for achievement motivation. Although the ICCs were modest, they indicated nonzero classroom-level dependence and supported the use of classroom-clustered inference for the adjusted GPA analyses. Inspection of standardized residuals, leverage values, and influence statistics indicated no additional cases exerting disproportionate influence on model estimates. Skewness and kurtosis values for primary variables fell within acceptable limits for regression-based analyses (absolute skewness < 1.20; absolute kurtosis <1.10), supporting approximate normality. Visual inspection of histograms and Q–Q plots further indicated no substantial deviations from linearity or homoscedasticity. Variance Inflation Factors (VIF) ranged from 1.08 to 1.46 across regression models, and tolerance values exceeded 0.68 in all cases, indicating no multicollinearity concerns. To assess the potential impact of common method variance, Harman’s single-factor test was conducted using unrotated exploratory factor analysis across all self-report items. The first unrotated factor accounted for 27.4% of total variance. This diagnostic does not rule out shared method variance; rather, it indicates only that no single common factor accounted for a dominant proportion of variance across the self-report items.

### 4.2 Measurement model

Confirmatory factor analyses were conducted separately for each multidimensional self-report measure using the correlated-factor structures specified in the Methods and Supplementary Table S1 described in the Methods and Supplementary Table S1. No items were deleted, no cross-loadings were estimated, and no residual covariances were added *post hoc*.

The three-factor model for dysregulated digital engagement demonstrated acceptable fit, χ^2^(167) = 271.16, *p* < 0.001, CFI = 0.94, TLI = 0.93, RMSEA = 0.045, 90% CI [0.035, 0.055], SRMR = 0.047. Standardized loadings ranged from 0.57 to 0.82.

The four-factor BPAQ model also demonstrated acceptable fit, χ^2^(371) = 612.79, *p* < 0.001, CFI = 0.92, TLI = 0.91, RMSEA = 0.046, 90% CI [0.039, 0.052], SRMR = 0.051. Standardized item loadings ranged from 0.51 to 0.84.

The three-factor achievement-motivation model demonstrated acceptable fit, χ^2^(347) = 544.61, *p* < 0.001, CFI = 0.94, TLI = 0.93, RMSEA = 0.043, 90% CI [0.036, 0.050], SRMR = 0.049. Standardized loadings ranged from 0.59 to 0.83.

These results support the intended multidimensional structures of the three measures. Importantly, the adequacy of the first-order factor models was not treated as sufficient evidence, by itself, for interpreting all dimensions as a single unidimensional construct. The scoring and analytic treatment of total and dimension-level scores are therefore justified separately in the revised analytic strategy (Table 2).

**Table 2:** Confirmatory factor analysis fit indices for study measures.

Measure	χ^2^ (df)	CFI	TLI	RMSEA	90% CI (RMSEA)	SRMR
Dysregulated digital engagement (3-factor)	271.16 (167)	0.94	0.93	0.045	[0.035, 0.055]	0.047
Aggression (4-factor BPAQ)	612.79 (371)	0.92	0.91	0.046	[0.039, 0.052]	0.051
Achievement motivation (3-factor)	544.61 (347)	0.94	0.93	0.043	[0.036, 0.050]	0.049

CFI = Comparative Fit Index; TLI = Tucker–Lewis Index; RMSEA = Root Mean Square Error of Approximation; SRMR = Standardized Root Mean Square Residual. All models were specified as correlated first-order simple-structure models with no item deletions, cross-loadings, or correlated residuals.

### 4.3 Descriptive statistics and reliability

Descriptive statistics, internal consistency coefficients, and distributional properties for the study variables are presented in Table 1.

Internal consistency coefficients ranged from 0.79 to 0.91, indicating satisfactory reliability across the self-report measures and subscales. GPA displayed slight negative skewness, although its distribution remained within the range considered acceptable for the regression analyses.

### 4.4 Zero-order correlations

Pearson correlations among regulatory difficulty, total aggression, the four aggression dimensions, achievement motivation, and GPA are reported in Table 3.

**Table 3:** Pearson correlations among study variables.

Variable	1	2	3	4	5	6	7	8
1. Regulatory difficulty	—							
2. Total aggression	0.38***	—						
3. Verbal aggression	0.40***	0.78***	—					
4. Physical aggression	0.25***	0.73***	0.46***	—				
5. Anger	0.31***	0.80***	0.52***	0.49***	—			
6. Hostility	0.29***	0.77***	0.50***	0.44***	0.58***	—		
7. Achievement motivation	−0.33***	−0.27***	−0.22***	−0.18**	−0.24***	−0.25***	—	
8. GPA	−0.28***	−0.18**	−0.16**	−0.13*	−0.15**	−0.17**	0.47***	—

*p < 0.05, **p < 0.01, ***p < 0.001.

Regulatory difficulty was positively associated with total aggression and with each of the four aggression dimensions. The numerically largest dimension-level correlation was observed for verbal aggression, followed by anger, hostility, and physical aggression. Regulatory difficulty was negatively associated with achievement motivation and GPA, whereas achievement motivation showed a positive association with GPA. Because the aggression dimensions are correlated and no formal pairwise comparison of dependent correlations was specified, the larger verbal-aggression coefficient is interpreted descriptively rather than as evidence of a statistically stronger association.

### 4.5 Associations of regulatory difficulty with aggression and achievement motivation

Separate raw-score linear regression models examined the association of regulatory difficulty with total aggression, achievement motivation, and each of the four aggression dimensions. Standardized and unstandardized coefficients were obtained from the same fitted models; Spearman coefficients from the bivariate analyses were not substituted for regression estimates (Table 4).

**Table 4:** Raw-score regression models relating regulatory difficulty to aggression and achievement motivation.

Outcome	*B*	SE	*β*	*t*	95% CI for B	*R* ^2^
Total aggression	0.865	0.120	0.380	7.20	[0.628, 1.101]	0.144
Verbal aggression	0.326	0.043	0.400	7.65	[0.242, 0.410]	0.160
Physical aggression	0.194	0.043	0.250	4.52	[0.110, 0.279]	0.062
Anger	0.248	0.043	0.310	5.71	[0.163, 0.334]	0.096
Hostility	0.221	0.042	0.290	5.31	[0.139, 0.303]	0.084
Achievement motivation	−0.691	0.113	−0.330	−6.13	[−0.913, −0.469]	0.109

B = unstandardized regression coefficient; SE = standard error; β = standardized regression coefficient; CI = confidence interval. All models were estimated using the original scored variables rather than rank-transformed variables. Unstandardized and standardized coefficients were derived from the same fitted models. Analyses involving Physical Aggression, Verbal Aggression, Anger, and Hostility were exploratory and were additionally evaluated using Benjamini–Hochberg false-discovery-rate control.

Regulatory difficulty was positively associated with total aggression and negatively associated with achievement motivation. Among the exploratory aggression dimensions, the numerically largest standardized association was observed for verbal aggression, followed by anger, hostility, and physical aggression. Because these coefficients were estimated for correlated aggression dimensions and were not subjected to formal pairwise tests of coefficient differences, the larger verbal-aggression estimate is interpreted descriptively rather than as evidence that its association was statistically stronger than those of the other aggression dimensions.

All four exploratory aggression-dimension associations remained statistically significant after Benjamini–Hochberg false-discovery-rate correction.

### 4.6 Secondary analyses of frequency and intensity

In secondary analyses, frequency showed a small positive association with total aggression (*β* = 0.190, *p* = 0.001) and a small negative association with achievement motivation (*β* = −0.120, *p* = 0.035). In the adjusted GPA model containing the same demographic and design-related covariates, total aggression, and achievement motivation as the primary model, frequency was not significantly associated with GPA (B = −0.033, robust SE = 0.028, *β* = −0.074, 95% CI [−0.095, 0.029], *p* = 0.238). Intensity showed somewhat larger associations with total aggression (*β* = 0.270, *p* < 0.001) and achievement motivation (*β* = −0.210, *p* < 0.001). Its adjusted association with GPA was negative but did not reach statistical significance (*B* = −0.055, robust SE = 0.032, *β* = −0.128, 95% CI [−0.125, 0.015], *p* = 0.081). These secondary findings suggest that the most consistent adjusted association with GPA was observed for regulatory difficulty rather than for the frequency or intensity dimensions.

### 4.7 Cluster-adjusted hierarchical regression predicting academic performance

A three-block hierarchical regression model was used to examine the incremental association of regulatory difficulty with GPA while accounting for the clustered sampling design. Age, gender, grade level, and school indicators were entered in Block 1 as demographic and design-related covariates. Total aggression and achievement motivation were added in Block 2, followed by regulatory difficulty in Block 3 as the focal digital-engagement predictor. Statistical inference for individual regression coefficients was based on classroom-clustered robust standard errors with a small-sample correction. Conventional *R*^2^ and Δ*R*^2^ values are reported descriptively to summarize explained variance across the three blocks (Table 5).

**Table 5:** Cluster-adjusted hierarchical regression predicting GPA.

Entry block	Predictor	*B*	Robust SE	*β*	95% CI for B	*p*
1	Age	−0.082	0.061	−0.037	[−0.217, 0.053]	0.205
1	Gender	0.214	0.183	0.051	[−0.189, 0.617]	0.268
1	Grade	−0.176	0.091	−0.071	[−0.377, 0.025]	0.078
1	School 2	0.292	0.221	0.064	[−0.194, 0.778]	0.214
1	School 3	−0.238	0.236	−0.049	[−0.758, 0.282]	0.336
2	Total Aggression	−0.018	0.007	−0.122	[−0.034, −0.002]	0.031
2	Achievement Motivation	0.064	0.012	0.397	[0.038, 0.090]	<0.001
3	Regulatory Difficulty	−0.072	0.027	−0.214	[−0.131, −0.013]	0.021

Model summary: Block 1 *R*^2^ = 0.087; Block 2 *R*^2^ = 0.304 (Δ*R*^2^ = 0.217); Block 3 *R*^2^ = 0.344 (Δ*R*^2^ = 0.040). B = unstandardized regression coefficient; Robust SE = classroom-clustered robust standard error; β = standardized regression coefficient; CI = confidence interval. School 1 served as the reference category. All coefficient estimates shown are from the final Block 3 model; the Entry block column indicates the stage at which each predictor was introduced. Conventional *R*^2^ and Δ*R*^2^ values are reported descriptively, whereas coefficient-level inference is based on small-sample-corrected classroom-clustered robust estimates. Regulatory Difficulty refers to the eight-item DE13–DE20 subscale.

The demographic and design-related covariates entered in Block 1 accounted for 8.7% of the descriptive variance in GPA (*R*^2^ = 0.087). Adding total aggression and achievement motivation in Block 2 increased *R*^2^ to 0.304 (Δ*R*^2^ = 0.217). Entry of regulatory difficulty in Block 3 increased the descriptive *R*^2^ to 0.344 (Δ*R*^2^ = 0.040). In the final cluster-adjusted model, regulatory difficulty was negatively associated with GPA after adjustment for the demographic and design-related covariates, total aggression, and achievement motivation (*B* = −0.072, robust SE = 0.027, *β* = −0.214, 95% CI [−0.131, −0.013], *p* = 0.021). Within the same final model, achievement motivation showed the largest absolute standardized association with GPA (*β* = 0.397), followed by regulatory difficulty (*β* = −0.214) and total aggression (*β* = −0.122).

### 4.8 Sensitivity analysis excluding academic-overlap items

To examine whether the association between regulatory difficulty and GPA was driven by direct overlap between predictor content and academic functioning, the final GPA model was repeated after excluding DE17–DE19, which explicitly referred to homework, studying, or school responsibilities. The resulting five-item regulatory-difficulty score, comprising DE13–DE16 and reverse-scored DE20, showed acceptable internal consistency (Cronbach’s *α* = 0.79). In the cluster-adjusted model, the reduced regulatory-difficulty score remained negatively associated with GPA after adjustment for the demographic and design-related covariates, total aggression, and achievement motivation (*B* = −0.085, robust SE = 0.037, *β* = −0.186, 95% CI [−0.166, −0.004], *p* = 0.031). The reduced score accounted for an additional 3.0% of descriptive variance in GPA (Δ*R*^2^ = 0.030), yielding a full-model *R*^2^ of 0.334. Compared with the primary analysis (*β* = −0.214, Δ*R*^2^ = 0.040), the association was modestly attenuated but remained statistically significant, indicating that the observed incremental association with GPA was not entirely attributable to the academically overlapping items.

## 5 Discussion

The present study examined the concurrent associations of regulatory difficulty in digital engagement with aggression, achievement motivation, and academic performance. Within the revised analytic model, greater regulatory difficulty was associated with higher aggression and lower achievement motivation and GPA. Importantly, regulatory difficulty retained a modest negative association with GPA after the measured demographic and design-related covariates, aggression, and achievement motivation were statistically accounted for. This result should be interpreted specifically as evidence of an incremental association within the present model. It does not establish that regulatory difficulty is psychometrically or conceptually independent of broader behavioral or motivational vulnerability, nor does it identify the processes responsible for the residual association. At the bivariate level, regulatory difficulty was positively associated with total aggression and with each of the four aggression dimensions. The numerically largest dimension-level association was observed for verbal aggression, followed by anger, hostility, and physical aggression. However, because the aggression dimensions are correlated and no formal pairwise comparisons of the dependent associations were conducted, these numerical differences should not be interpreted as evidence that regulatory difficulty is preferentially or uniquely associated with verbal aggression. Moreover, the present digital-engagement measure does not distinguish among specific forms of online activity, and the aggression measure assesses general rather than specifically online aggression. Activity-specific studies are therefore needed to determine whether particular digital contexts are differentially associated with verbal or other forms of aggressive behavior. The observed negative association between dysregulated engagement and achievement motivation similarly aligns with self-determination and self-regulation frameworks. Achievement motivation depends upon persistence, sustained effort, and tolerance of delayed reinforcement. Digital platforms, by contrast, often operate through immediate feedback loops and intermittent reinforcement schedules. Difficulty disengaging from highly salient digital activities may be associated with greater orientation toward immediate rewards relative to longer-term academic goals. Such an interpretation is theoretically plausible, but reward sensitivity and self-regulatory capacity were not directly measured in the present study and therefore cannot be identified as mechanisms underlying the observed association with achievement motivation. The magnitude of the association, while moderate, is consistent with multifactorial models of academic development in which no single predictor dominates but multiple influences converge.

A central result concerns the incremental association between regulatory difficulty and GPA. After the demographic and design-related covariates, total aggression, and achievement motivation were taken into account, regulatory difficulty was associated with a modest additional proportion of explained variance in academic performance. This finding indicates that regulatory difficulty contained statistical information about GPA that was not fully shared with the predictors included in this particular model. However, statistical increment should not be equated with construct validation or conceptual independence. The present analyses cannot determine whether regulatory difficulty represents a distinct underlying psychological process, because other unmeasured characteristics could account for part of the remaining association.

One possibility for future investigation is that difficulty disengaging from digital environments may be associated with attentional allocation or executive-control processes relevant to academic work. For example, frequent interruption or difficulty shifting attention away from highly salient digital activities could plausibly interfere with sustained academic concentration. However, attention and executive functioning were not measured in the present study, and the current findings therefore provide no direct test of this account. Such processes should be regarded as hypotheses for future longitudinal or experimentally informed research rather than as mechanisms established by the present data.

It is equally important to interpret these findings with conceptual restraint. The cross-sectional design does not permit causal or temporal inference. Greater regulatory difficulty was associated with lower academic performance, but the direction of this association cannot be determined from the present data. Academic difficulties may precede dysregulated digital engagement, dysregulated engagement may precede poorer academic functioning, or both may develop reciprocally or reflect other unmeasured factors. Longitudinal and cross-lagged designs will be necessary to evaluate these alternatives. Future longitudinal studies could extend the present analysis using structural equation or cross-lagged path models to test theoretically specified direct and indirect relationships among digital engagement, behavioral and motivational variables, and academic outcomes. Accordingly, the present results should be interpreted as concurrent and incremental statistical associations within the measured variable set rather than as evidence of a specific developmental pathway. The integration of aggression and motivation within a single analytic framework represents an additional contribution. Much of the prior literature has examined digital engagement in isolation or in relation to a single outcome domain. By modeling aggression, achievement motivation, and GPA within the same analytic framework, the present study clarifies their concurrent and incremental statistical associations with regulatory difficulty. The strong positive association between achievement motivation and GPA replicates established findings and reinforces the centrality of motivational processes in academic success. The weaker but significant negative association between aggression and GPA is consistent with research linking externalizing behaviors to classroom adjustment challenges. Against this backdrop, the incremental association of regulatory difficulty indicates that it accounted for additional statistical variance in GPA beyond the measured aggression and achievement-motivation scores, without implying that these constructs are psychologically independent.

The present findings also add evidence from a Chinese secondary-school context to a literature in which digital behavior has been operationalized in diverse ways. Previous studies in Chinese samples have examined constructs such as mobile-phone addiction and social-media use in relation to school functioning (29, 30). The present study extends this contextual evidence by examining dysregulated digital engagement together with aggression, achievement motivation, and school-recorded GPA. However, the sampling of three schools in eastern China does not constitute a cross-cultural comparison, and the present data cannot determine whether the observed associations are specific to Chinese educational or cultural conditions. Academic pressure, parental expectations, and related contextual characteristics were not directly assessed. These findings should therefore be interpreted as evidence obtained within a Chinese context rather than evidence of a specifically Chinese or cross-cultural mechanism. The findings may also inform future educational and intervention research, although the present cross-sectional study does not establish the effectiveness of any specific intervention strategy. Rather than treating exposure duration and regulatory characteristics as competing explanations, future studies could assess both simultaneously and examine whether approaches targeting goal setting, time management, or digital self-regulation are associated with improved academic functioning. Such intervention-oriented questions require prospective evaluation before recommendations regarding comparative effectiveness can be made.

At the same time, several limitations warrant careful consideration. First, the reliance on self-report measures for digital engagement, aggression, and motivation introduces the possibility of response bias, despite procedural steps to mitigate common method variance. Although Harman’s single-factor test suggested that shared method variance was unlikely to account for the majority of covariance, future research would benefit from incorporating behavioral or observational indices of digital use, such as device-logged data. Second, the cross-sectional design precludes inference regarding temporal ordering or causality. Longitudinal designs are essential for evaluating developmental trajectories and reciprocal influences. In addition, general self-regulatory capacity was not directly measured; therefore, the study cannot determine whether self-regulation explains the observed associations among digital engagement, aggression, motivation, and academic performance. The clustered sampling design also warrants consideration. Although the revised analyses account for classroom clustering using small-sample-corrected cluster-robust inference and adjust for school and grade, the sample was drawn from only three schools. Consequently, school-level contextual effects cannot be estimated with precision, and generalization to other educational settings should remain cautious. The clustered design also reduces the effective information available relative to an equally sized simple random sample, and the study may therefore have had limited sensitivity for detecting very small incremental effects despite the nominal sample size. Although age, gender, grade level, and school membership were included in the adjusted GPA model, several potentially relevant covariates were not measured, including socioeconomic status, parental education, sleep quality, study habits, and broader aspects of the family environment. These unmeasured factors may be associated with both digital-engagement patterns and academic performance and could therefore account for part of the observed association. The incremental coefficient for regulatory difficulty should consequently be interpreted as conditional on the variables included in the present model rather than as an estimate free from residual confounding. Third, the operationalization of dysregulated engagement, while multidimensional and supported by confirmatory factor analysis, does not distinguish among specific types of digital activity. Different forms of online engagement—educational, social, gaming, or passive consumption—may exhibit distinct associations with regulatory processes and academic outcomes. Future research should incorporate content differentiation to refine theoretical precision.

Finally, the study was not preregistered, and the distinction between primary, secondary, exploratory, and sensitivity analyses should therefore be interpreted in light of the absence of a time-stamped preregistered analysis plan.

Another consideration concerns effect magnitude. While statistically significant, the incremental contribution of dysregulated engagement to GPA was modest. This should not be interpreted as evidence of negligible importance but rather as consistent with the complexity of academic achievement. In multifactorial systems, small effects can accumulate and interact over time. Nonetheless, replication across diverse samples and educational contexts is necessary to evaluate generalizability. Despite these limitations, the present study adds evidence on the concurrent relationships among regulatory difficulty, aggression, achievement motivation, and academic performance. The incremental GPA association remained after the measured behavioral and motivational variables were statistically accounted for, but its modest magnitude and the cross-sectional design require cautious interpretation. Regulatory difficulty should therefore be viewed as one correlate within a broader set of factors associated with adolescent academic functioning rather than as an independently validated mechanism. As digital environments continue to evolve, understanding patterns of regulated and dysregulated engagement remains an important direction for developmental research. Future studies that directly assess self-regulatory, attentional, and executive processes will be necessary to determine whether these mechanisms help explain the associations observed here. Examining regulatory characteristics alongside exposure-related measures may provide a more differentiated account of adolescent functioning in increasingly digital environments.

## 6 Conclusion

The present study found that greater regulatory difficulty in digital engagement was associated with higher aggression, lower achievement motivation, and lower GPA among adolescents. Regulatory difficulty also retained a modest negative association with GPA after demographic and design-related covariates, total aggression, and achievement motivation were statistically accounted for. These findings should be interpreted as concurrent and incremental associations rather than evidence of causal or self-regulatory mechanisms. The results support continued investigation of regulatory characteristics of digital engagement alongside conventional exposure measures, particularly using longitudinal designs and direct measures of the psychological processes proposed to underlie these associations.

## Data Availability

The original contributions presented in the study are included in the article/Supplementary material, further inquiries can be directed to the corresponding author/s.
