# Supplementary material for: When digital engagement becomes dysregulated: associations with aggression, achievement motivation, and academic performance in adolescents
Source: Front Public Health. 2026 Sep 3;14:1896602. doi: 10.3389/fpubh.2026.1896602 (PMC13581765; doi:10.3389/fpubh.2026.1896602)
Supplement: Supplementary file 1 [file Data_Sheet_1.pdf]

Supplementary Material

When Digital Engagement Becomes Dysregulated: Associations with Aggression, Achievement Motivation, and Academic Performance in Adolescents

Supplementary Measurement Information

To improve transparency and reproducibility, Supplementary Table S1 provides detailed information on the composition and scoring of the self-report measures used in the study. For the two study-adapted measures—dysregulated digital engagement and achievement motivation—the table reports the complete item wording, dimensional assignment, scoring direction, and conceptual basis for item development. For the Buss–Perry Aggression Questionnaire (BPAQ), item wording is not reproduced in full; instead, the table reports the original item numbering, subscale allocation, scoring direction, and source citation. This approach provides sufficient information to reconstruct the scoring procedures while respecting the reproduction conditions of the established instrument. Items of the digital-engagement measure that explicitly refer to homework, studying, or school responsibilities are additionally identified because they were excluded in the content-overlap sensitivity analysis predicting GPA. The digital-engagement measure should therefore be interpreted as a study-adapted instrument evaluated psychometrically in the present sample rather than as an independently validated standardized scale.

Supplementary Table S1. Item composition, dimensional allocation, and scoring procedures for the self-report measures used in the study.

A. Dysregulated Digital Engagement — 20 items

Item	Dimension	Item wording	Scoring	Adaptation basis	Academic-overlap item
DE1	Frequency	I check online platforms many times throughout the day.	Direct	Frequency of digital engagement	No
DE2	Frequency	Going online is one of the activities I do most frequently during my free time.	Direct	Frequency of digital engagement	No
DE3	Frequency	I repeatedly check digital platforms even when I have no specific reason to do so.	Direct	Habitual checking/frequency	No
DE4	Frequency	I use online platforms at many different times during a typical day.	Direct	Frequency of engagement	No
DE5	Frequency	I return to online activities shortly after I	Direct	Repetitive engagement	No

Item	Dimension	Item wording	Scoring	Adaptation basis	Academic-overlap item
		have stopped using them.			
DE6	Frequency	I spend parts of most days engaged with online platforms or digital applications.	Direct	Frequency of engagement	No
DE7	Intensity	Online activities often hold my attention for longer than I originally intended.	Direct	Salience/intensity	No
DE8	Intensity	I become deeply absorbed in what I am doing online.	Direct	Psychological involvement	No
DE9	Intensity	Online activities often feel more engaging than other things happening around me.	Direct	Salience/involvement	No
DE10	Intensity	I often continue an online activity because I do not want to interrupt it.	Direct	Sustained involvement	No
DE11	Intensity	I frequently lose track of time while using online platforms.	Direct	Absorption/intensity	No
DE12	Intensity	Digital activities can become the main focus of my attention for long periods.	Direct	Salience/intensity	No
DE13	Regulatory difficulty	I find it difficult to stop using online platforms even when I know I should.	Direct	Impaired control/difficulty disengaging	No
DE14	Regulatory difficulty	I have tried to reduce my online activity but found it difficult to do so.	Direct	Impaired control	No
DE15	Regulatory difficulty	I feel restless or uncomfortable when I cannot access the internet.	Direct	Withdrawal-like discomfort	No
DE16	Regulatory difficulty	I sometimes continue using digital platforms even when I would prefer to stop.	Direct	Loss of control	No
DE17	Regulatory difficulty	My online activities interfere with my homework or study time.	Direct	Functional interference	**Yes**
DE18	Regulatory difficulty	I sometimes stay online even when I know I should be studying.	Direct	Academic interference/impaired control	**Yes**
DE19	Regulatory difficulty	My digital activities sometimes make it harder for me to complete school responsibilities on time.	Direct	Academic interference	**Yes**
DE20	Regulatory difficulty	I can usually stop using online platforms whenever I decide to do so.	**Reverse**	Perceived regulatory control	No

B. Aggression — BPAQ

Construct	Subscale	Original BPAQ item numbers	No. of items	Scoring	Source
Aggression	Physical Aggression	2, 5, 8, 11, 13, 16, 22, 25, 29	9	Items scored 1–5; item 16 reverse scored	Buss & Perry (1992)
Aggression	Verbal Aggression	4, 6, 14, 21, 27	5	Items scored 1–5	Buss & Perry (1992)
Aggression	Anger	1, 9, 12, 18, 19, 23, 28	7	Items scored 1–5; item 9 reverse scored	Buss & Perry (1992)
Aggression	Hostility	3, 7, 10, 15, 17, 20, 24,	8	Items scored 1–5	Buss & Perry

Construct	Subscale	Original BPAQ item numbers	No. of items	Scoring	Source
		26			(1992)
Aggression	Total aggression	All 29 items	29	Sum of the four subscales after reverse scoring	Buss & Perry (1992)

C. Achievement Motivation — 28 items

Item	Dimension	Item wording	Scoring	Conceptual basis
AM1	Persistence	I continue working on difficult school tasks even when they are challenging.	Direct	Persistence
AM2	Persistence	I keep trying when I do not understand something immediately.	Direct	Persistence
AM3	Persistence	I usually finish academic tasks that I have started.	Direct	Persistence
AM4	Persistence	I continue working toward academic goals even after setbacks.	Direct	Persistence
AM5	Persistence	I am willing to spend a long time solving a difficult academic problem.	Direct	Persistence
AM6	Persistence	I tend to give up when schoolwork becomes difficult.	**Reverse**	Persistence
AM7	Persistence	I can remain focused on a challenging task until I make progress.	Direct	Persistence
AM8	Persistence	When my first attempt fails, I usually try another way to solve the problem.	Direct	Persistence
AM9	Persistence	I keep working even when success requires repeated effort.	Direct	Persistence
AM10	Persistence	Difficult assignments often make me stop trying.	**Reverse**	Persistence
AM11	Effort investment	I put substantial effort into doing well at school.	Direct	Effort
AM12	Effort investment	I work hard even when an assignment is not especially interesting.	Direct	Effort
AM13	Effort investment	I prepare carefully for important school tasks and examinations.	Direct	Effort
AM14	Effort investment	I try to improve the quality of my schoolwork whenever possible.	Direct	Effort
AM15	Effort investment	I devote enough time to academic tasks that are important to me.	Direct	Effort
AM16	Effort investment	I often do only the minimum amount of work required at school.	**Reverse**	Effort
AM17	Effort investment	I am willing to make extra effort when a subject is difficult.	Direct	Effort
AM18	Effort investment	Doing well in school is important enough for me to work hard for it.	Direct	Effort/goal pursuit
AM19	Effort investment	I frequently postpone schoolwork even when I have enough time to complete it.	**Reverse**	Effort regulation
AM20	Task orientation	I feel satisfied when I master a difficult academic task.	Direct	Mastery/task orientation
AM21	Task orientation	Learning something thoroughly is more important to me than simply finishing quickly.	Direct	Task orientation
AM22	Task	I enjoy improving my ability in subjects that require effort.	Direct	Mastery orientation

Item	Dimension	Item wording	Scoring	Conceptual basis
	orientation			
AM23	Task orientation	I set specific goals for improving my academic work.	Direct	Goal-directed task orientation
AM24	Task orientation	I pay attention to whether I am making progress in my schoolwork.	Direct	Task orientation
AM25	Task orientation	I try to understand my mistakes so that I can perform better next time.	Direct	Mastery orientation
AM26	Task orientation	I prefer school tasks that allow me to develop my abilities.	Direct	Task orientation
AM27	Task orientation	I am mainly interested in completing schoolwork with as little effort as possible.	**Reverse**	Low task orientation
AM28	Task orientation	Improving my knowledge and skills is an important reason for doing schoolwork.	Direct	Mastery/task orientation

Note. DE=dysregulated digital engagement; AM=achievement motivation; BPAQ=Buss–Perry Aggression Questionnaire. The dysregulated-digital-engagement and achievement-motivation measures were study-adapted instruments informed by established conceptual and measurement traditions rather than unmodified administrations of a single standardized questionnaire. All items were rated on five-point response scales. For the digital-engagement measure, responses ranged from 1 (strongly disagree) to 5 (strongly agree); DE20 was reverse scored before calculation of dimension and total scores. Frequency comprised DE1–DE6, intensity comprised DE7–DE12, and regulatory difficulty comprised DE13–DE20. DE17–DE19 explicitly refer to homework, studying, or school responsibilities. Accordingly, the primary regulatory-difficulty analysis predicting GPA was repeated after excluding these three items. The resulting content-overlap sensitivity score comprised the remaining five regulatory-difficulty items (DE13–DE16 and DE20, with DE20 reverse scored).

For achievement motivation, responses ranged from 1 (strongly disagree) to 5 (strongly agree). AM6, AM10, AM16, AM19, and AM27 were reverse scored. Persistence comprised AM1–AM10, effort investment comprised AM11–AM19, and task orientation comprised AM20–AM28. Higher scores indicated greater achievement motivation.

Aggression was scored according to the four-domain structure of the 29-item Buss–Perry Aggression Questionnaire (Buss & Perry, 1992): Physical Aggression (9 items), Verbal Aggression (5 items), Anger (7 items), and Hostility (8 items). BPAQ items 9 and 16 were reverse scored before calculating subscale and total scores. Full BPAQ item wording is not reproduced here; readers are referred to the original publication for the instrument content. Higher scores on all aggression scales indicate greater aggression.

Scoring and analytic use

For the primary analyses, the regulatory-difficulty dimension of digital engagement was used as the focal digital predictor, together with total aggression and total achievement motivation. Frequency and intensity were retained as separate secondary dimensions and were not combined with regulatory difficulty in the principal hypothesis tests. Dimension-level aggression analyses were treated as exploratory. Because DE17–DE19 explicitly refer to homework, studying, or school responsibilities, the GPA model was additionally repeated using a reduced five-item regulatory-difficulty score comprising DE13–DE16 and DE20. The reduced score showed acceptable internal consistency (Cronbach's α=.79), and the corresponding sensitivity-analysis estimates are reported in the revised Results section.
